# Replacing fishmeal with plant protein in Atlantic salmon (*Salmo salar*) diets by supplementation with fish protein hydrolysate

**DOI:** 10.1038/s41598-020-60325-7

**Published:** 2020-03-06

**Authors:** S. Egerton, A. Wan, K. Murphy, F. Collins, G. Ahern, I. Sugrue, K. Busca, F. Egan, N. Muller, J. Whooley, P. McGinnity, S. Culloty, R. P. Ross, C. Stanton

**Affiliations:** 1 School of Microbiology, University College Cork, Cork, Ireland; 2 School of Biological, Earth and Environmental Sciences, University College Cork, Cork, Ireland; 3 APC Microbiome Ireland, Cork, Ireland; 4 Aquaculture Nutrition and Aquafeed Research Unit (ANARU), Carna Research Station, Ryan Institute, NUI Galway, Galway, Ireland; 5 Teagasc Food Research Centre, Moorepark, Fermoy, Co. Cork, Ireland; 6 Biomarine Ingredients Ireland Ltd., Monaghan, Ireland; 7 Environmental Research Institute, University College Cork, Cork, Ireland

**Keywords:** Metagenomics, Ichthyology

## Abstract

The effects of feeding an 80% plant protein diet, with and without fish protein hydrolysate (FPH) supplementation, on the growth and gut health of Atlantic salmon were investigated. Fish were fed either (A) a control diet containing 35% fishmeal, (B) an 80% plant protein diet with 15% fishmeal, (C) an 80% plant protein diet with 5% fishmeal and 10% partly hydrolysed protein, or (D) an 80% plant protein diet with 5% fishmeal and 10% soluble protein hydrolysate. Fish on the 80% plant- 15% fishmeal diet were significantly smaller than fish in the other dietary groups. However, partly-hydrolysed protein supplementation allowed fish to grow as well as fish fed the control 35% fishmeal diet. Fish fed the FPH diets (diets C and D) had significantly higher levels of amino acids in their blood, including 48% and 27% more branched chain amino acids compared to fish on the 35% fishmeal diet, respectively. Plant protein significantly altered gut microbial composition, significantly decreasing α-diversity. Spirochaetes and the families *Moritellaceae, Psychromonadaceae, Helicobacteraceae* and *Bacteroidaceae* were all found at significantly lower abundances in the groups fed 80% plant protein diets compared to the control fishmeal diet.

## Introduction

Fishmeal is generally considered the gold standard dietary protein source for many fish species. Its production is based upon wild marine fish of no commercial value^1^. However, today it is considered both environmentally and ecologically unsustainable and there are societal and economic pressures on the aquaculture industry to find alternative proteins. Removing fishmeal from the diets of omnivorous species has been readily achieved, but this has been more difficult to implement in carnivorous fish and crustaceans^2^. It has been generally found that up to 50% fishmeal protein can be replaced by plant proteins in carnivorous fish diets without any negative effects on growth or fish welfare issues^3^. Plant proteins are the most common replacements for fishmeal in aquafeeds. They are cost-effective and are often preferred because of the negative consumer perception around the use of terrestrial animal by-products for feeding fish^4–6^. However, even when aquafeeds high in plant protein ( > 50%) are formulated to provide the required balance of amino acids and other essential nutrients (e.g. fatty acids, macro and trace metals), the growth performance obtained is inferior to that of fish fed fishmeal-based diets^2,4,5,7^. These shortcomings are often the result of plant proteins possessing anti-nutritional factors (e.g. phytate, saponins, lectins) and indigestible carbohydrates, as well as less efficient protein digestion and amino acid absorption^8^. It has also been shown that different dietary protein sources alter fish gut microbiota^4,9^ and such changes have, at times, been linked to subsequent deterioration in health, immunity and growth^10–14^.

There has been considerable research on re-formulating aquafeeds using novel ingredients and nutritional supplements (e.g. exogenous enzymes, bioactive compounds and bioavailable trace metals) that complement plant proteins and help to meet the needs of aquaculture species^15–18^. Creating sustainable feeds that promote fish welfare and maximise growth potential, while remaining cost-efficient, is a prominent challenge for the aquaculture industry. Furthermore, it is now recognised that the effects of dietary alterations on the gut microbiota of fish must be considered, as they play a key role in influencing fish health and growth^11,19,20^. Some promising work is emerging to suggest that diets with very low or no fishmeal inclusion will be possible with careful formulation in the future^21,22^. However, to date, it has been recommended that for optimal growth a minimum of 5% fishmeal is required to provide unidentified growth factors, thought to be naturally occurring trace and ultra-trace compounds such as amines and steroids^3,23^.

While a detailed understanding of the composition and structure of the intestinal microbiota in Atlantic salmon is still developing^11^, several studies have investigated the effects of alternative protein sources and high plant-protein/ low fishmeal diets^4,10,24,25^. The reported changes linked to inclusion of dietary plant proteins have varied but there are also some interesting similarities emerging. In one study it was found that lactic acid bacteria increased in fish fed plant-based diets^4^. Hartviksen *et al*.^10^, comparing more than one type of plant-protein source, found that extracted sunflower meal caused an increase in the relative abundance of *Lactobacillaceae* (and *Corynebacteriaceae*) but reduced *Streptococcaceae* and *Peptostreptococcaceae*, while pea protein concentrate resulted in a significant increase in *Vibrionaceae*. Recent findings from a study investigating fishmeal-free diets reported higher levels of *Streptococcus* spp. associated with fishmeal diets and *Lactobacillus* spp. associated with the alternative protein diet^25^. In terms of pathogenesis associated with high plant protein diets, increased relative abundance of *Psychrobacter* spp., *Enterococcus* spp., *Micrococcus* spp., and *Staphylococcus* spp. have been proposed^26^. These studies were all carried out on adult Atlantic salmon. Few studies have investigated the gut microbiota at earlier life stages^27,28^, and none of these studies have investigated the effects of alternative dietary protein sources on the gut microbiota. The results from these studies, however, have found significant differences in the gut microbiota composition from those reported for Atlantic salmon at sea^29,30^.

Fish protein hydrolysates (FPH) are products from either chemical (e.g. acid and alkaline) or enzymatic (e.g. protease) breakdown of fish proteins into single amino acids, peptides and oligopeptides^31^. High quality FPH can be produced from fish processing by-products, fishery by-catch, and low-value pelagic species not currently directly consumed by humans^32,33^. They are considered a suitable source of protein for human and animal nutrition because of their balanced amino acid composition and their low molecular weight, allowing higher gut absorption rates^32,34^. Their addition at low concentrations (18–24%) has been found to significantly increase individual specific growth rates of adult Atlantic salmon (*Salmo salar*)^35^. More recently, Atlantic salmon, at the fast-growing seawater stage, were found to grow equally well on a diet consisting of plant proteins supplemented with 5% fishmeal, 5% fish soluble protein and 3% squid hydrolysate as on a fishmeal control diet^23^. The supplemented fish-derived fractions of this diet increased palatability and provided sufficient bioavailable nutrients to compensate for the nutritional shortcomings of the plant protein ingredients (e.g. antinutrients and lower bioavailability of nutrients)^23^.

FPH, added at appropriate levels, has been reported to increase survival and growth rates, decrease malformation rates, increase enzyme activity, modify nutrient transport patterns in the intestine, improve nutrient absorption and induce non-specific immune responses in larvae, fry and adult fish^36–41^. FPH added to Atlantic salmon diets have resulted in positive immune modulation (enhanced levels of superoxide anion production in head kidney leucocytes) in adult fish^36^ and increased feed intake (12.5% greater than control) and growth (1.8% higher specific growth rate compared to control) in post-smolts^35^.

In this study, a 12-week feeding trial, we investigated the effects of high plant-protein/low fishmeal diets, with and without FPH supplementation on growth performance and gut health in Atlantic salmon parr, on-grown in freshwater. Only two previous studies conducted in the past 25 years have investigated the effects of FPH supplementation on growth performance in juvenile freshwater Atlantic salmon, neither of which used plant protein-based diets^42,43^. Freshwater juvenile Atlantic salmon have much higher growth rates compared to seawater and post-smolt salmon^44^. Furthermore, it is important for juveniles to have a high nutritional status and energy turnover in order to successfully undergo smoltification and a generally positive relationship between salmon smolt size and survival is frequently noted^45,46^. Thus, there is a high requirement for aquafeeds, focussed towards this life stage, to provide optimal growth and fish health while meeting market demands in sustainability.

## Results

### Growth performance, feed intake and biometric measures

Fish were fed either (A) a control diet containing 35% fishmeal (FM), (B) an 80% plant protein diet supplemented with 15% fishmeal (PL), (C) an 80% plant protein diet supplemented with 5% fishmeal and 10% partly hydrolysed FPH (PHP), or (D) an 80% plant protein diet supplemented with 5% fishmeal and 10% soluble FPH (SPH; Table 1). Feed utilisation and conversion measures were calculated from tank averages (Table 2). Feed intake was similar for all tanks and ranged from 24.81–27.43 g fish^−1^. Apparent digestibility coefficient of dry matter was greatest for FM and SPH groups (80.83 and 80.00%) and notably lower in fish fed the PL diet (70.77%). The results of the apparent digestibility are not unexpected; however, they must be considered with caution because multiple analyses were not possible. Low volumes of faecal matter after drying meant that samples had to be pooled for apparent digestibility analyses. Specific growth rates were highest for FM and PHP fish and lowest for PL fish. Similarly, PHP and FM fish showed the highest protein productive value and protein efficiency ratio, while SPH fish had the lowest. Conversely, feed conversion ratio was lowest for PHP followed by FM fish. The preceeding observed differences were not found to be statistically significant. Hepatosomatic index, however, was significantly higher in the PHP fish compared to PL fish (F = 4.469, df = 3, *p* < 0.05; Table 2).

**Table 1:** Treatment diets formulation and proximate composition (%, dry matter), and estimated costs of variable protein ingredients (€/metric tonnes (MT)).

Diet Formulation	FM	PL	PHP	SPH
Fishmeal^a^	35.00	15.00	5.00	5.00
Soy meal concentrate^b^	14.34	40.33	40.16	35.68
PHP^c^	—	—	10.00	—
SPH^c^	—	—	—	10.00
Fish oil^a^	14.00	14.00	14.00	14.00
Wheat gluten^d^	9.00	9.00	9.00	9.00
Pea protein^d^	9.00	9.00	9.00	9.00
Rape seed oil^e^	1.18	3.29	3.14	4.29
Potato starch^f^	13.98	5.88	6.20	9.53
Vitamin & mineral premix^g^	2.00	2.00	2.00	2.00
Lysine^h^	0.50	0.50	0.50	0.50
Antioxidants^i^	0.50	0.50	0.50	0.50
Methionine^h^	0.30	0.30	0.30	0.30
Molasses^j^	0.20	0.20	0.20	0.20
**Proximate Composition**				
Moisture	5.54	5.25	5.8	5.91
Crude protein	47.78	47.67	47.73	46.46
Crude lipid	19.22	19.46	19.29	19.97
Ash	8.02	5.68	4.64	4.86
**Cost Comparison (€/MT Feed)**				
Fishmeal^k^	456.17	195.50	65.17	65.17
Soy meal concentrate^l^	84.41	237.40	236.40	210.03
PHP^c^	—	—	240.00	—
SPH^c^	—	—	—	400.00
Total	540.58	432.90	541.56	675.19

^a^United Fish Industries, Grimsby, UK.

^b^HP100, Hamlet proteins, Horsens, Denmark

^c^Biomarine Ingredients Ireland, Monaghan, Ireland.

^d^Roquette Freres, Lestrem, France.

^e^KTC Edibles Ltd., Wednesbury, UK.

^f^Terros, Origny-Sainte-Benoite, France.

^g^Premier Nutrition Products Ltd., UK. Manufacturer analysis: Ca 12.09%, Ash 78.71%, Na 8.86%, Vitamin A1.0 μg/kg, Vitamin D 0.10%, Vitamin E 7.0 g/kg, Cu 250 mg/kg and P 5.2 g/kg.

^h^Biomar Ltd., Northshore Road, Grangemouth Docks, Scotland.

^i^Barox plus liquid, Kemin Europa N.V., Belgium.

^j^Target Baits, Whitchurch, Shropshire, UK.

^k^Indexmundi.com: World Bank, March 2019.

^l^Alibaba.com: Agrosul Agroavicola Industrial SA, Brazil, April 2019.

**Table 2:** The feed utilisation and growth performance of Atlantic salmon parr fed four different experimental diets (FM, PL, PHP and SPH diets) for 12 weeks. Values with different superscripts in the same row are significantly different (^†^=one-way ANOVA; *p* < 0.05, *n*=3, ± SD, *=Kruskal-Wallis H test and Duncan’s Multiple Comparison test; *p* < 0.05, ± SD).

	FM	PL	PHP	SPH
Mortalities	**11**	**10**	**13**	**6**
Apparent digestibility;%	**80.83**	**70.77**	**72.86**	**80.00**
Feed intake; g/fish^†^	**26.55** ± 1.17	**25.44** ± 1.88	**24.81** ± 1.03	**27.43** ± 0.50
Feed conversion ratio^†^	**0.92** ± 0.09	**1.04** ± 0.06	**0.88** ± 0.07	**1.05 **± 0.09
Protein production value^†^	**3.34** ± 0.43	**2.67** ± 0.19	**3.08** ± 0.21	**2.52** ± 0.19
Protein efficiency ratio^†^	**2.48** ± 0.26	**2.21** ± 0.13	**2.57** ± 0.13	**2.19 **± 0.20
Liver weight; mg^†^	**499.67** ± 17.73^ab^	**425.29** ± 25.57^a^	**561.67** ± 28.11^b^	**484.21** ± 30.29^ab^
Hepatosomatic Index;%^†^	**1.31** ± 0.03^ab^	**1.25** ± 0.12^a^	**1.52 **± 0.08^b^	**1.35** ± 0.05^ab^
Final weight; g*	**38.13** ± 2.84^a^	**34.14** ± 3.23^b^	**37.03** ± 0.88^c^	**35.71** ± 1.77^bc^
Final length; cm*	**14.39** ± 0.34 ^a^	**13.87** ± 0.37^b^	**13.92** ± 0.03^b^	**14.00** ± 0.35^b^
Final condition factor; k*	**1.23** ± 0.01 ^a^	**1.24** ± 0.04 ^a^	**1.32** ± 0.03^b^	**1.26** ± 0.04^c^

The principle fish growth performance indicators of the feeding trial were individual weight, fork length and condition factor at the end of the 12-week feeding period. Average weight gain of treatment groups after the 12-week feeding trial was between 34.14 and 38.13 g. FM, PHP and SPH diets were found to produce significantly heavier fish than the PL diet. Fish on the FM diet were also found to be significantly heavier than fish on the SPH diet (χ^2^ = 59.237, df = 3, *p* < 0.001) but did not differ significantly to PHP fed fish (Table 2). Fish in the group fed the FM diet were significantly longer than all other groups (χ^2^ = 45.045, df = 3, *p* < 0.001, Table 2). Condition factor was calculated using the fork length and total weight of each individual fish. Fish on the PHP diet had a condition factor of 1.32, which was significantly greater than all other fish (χ^2^ = 144.217, df = 3, *p* < 0.001, Table 2).

### Proximate composition

The whole-body proximate compositions of fish before (pre-treatment, PT) and after the dietary treatment were analysed (Table 3). Water content in fish sampled from all groups was not significantly different (F = 1.786, df = 4, *p* = 0.145). Of the treated fish, the FM group had significantly higher ash content (2.14 ± 0.05%) compared to the fish on the other treatments (1.59 ± 0.04–1.74 ± 0.03%; F = 77.441, df = 4, *p* < 0.001). The lipid content of fish on the SPH treatment was significantly reduced (9.55 ± 0.25%) compared to that of the pre-treatment (PT) fish (10.96 ± 0.27%; F = 4.495, df = 4, *p* < 0.01). Finally, protein content in FM fish (17.26 ± 0.13%) was significantly greater than that of fish on the SPH treatment (16.76 ± 0.10%; F = 2.615, df = 4, *p* < 0.05, Table 3).

**Table 3:** The proximate compositions of salmon parr before dietary treatment (PT) and after 12 week feeding trial with four different experimental diets (FM, PL, PHP and SPH diets). Values with different superscripts in the same row are significantly different (one-way ANOVA; *p* < 0.05, *n* = 3, ± SD).

	PT	FM	PL	PHP	SPH
Moisture	**72.16** ± 3.26	**70.43** ± 3.15	**72.81** ± 3.67	**72.12** ± 3.17	**74.24** ± 3.76
Protein	**17.04** ± 0.47^ab^	**17.26** ± 0.43^a^	**16.82** ± 0.23^ab^	**17.07** ± 0.57^ab^	**16.76** ± 0.32^b^
Lipid	**10.96** ± 0.89^a^	**10.31** ± 0.67^ab^	**10.14** ± 0.78^ab^	**10.07** ± 0.79^ab^	**9.55** ± 0.81^b^
Ash	**2.46** ± 0.18^a^	**2.14** ± 0.18^b^	**1.74** ± 0.11^c^	**1.58** ± 0.12^c^	**1.63** ± 0.10^c^

**Figure 1:**
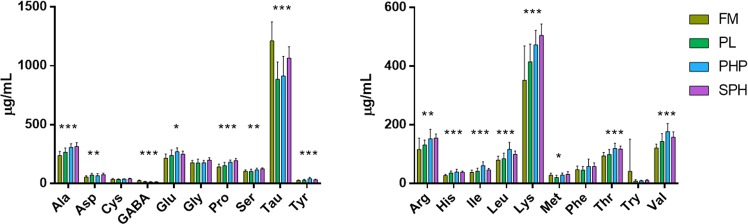
Blood amino acid concentrations. (**a**) Dispensable and (**b**) indispensable blood free amino acid levels in fish fed one of four diets; FM, PL, PHP or SPH. Values are means ± SD (*n* = 11). Statistical analysis was completed using one-way ANOVA and Tukey’s Multiple Comparison test. Significant differences denoted as: *(*p* < 0.05), **(*p* < 0.01), and ***(*p* < 0.001).

While total protein content only differed significantly between the two groups (FM and SPH), free amino acid concentrations in the blood samples showed multiple significant differences. Total free amino acids ranged from 3000 to 3534 µg mL^−1^ in the blood samples. SPH fish had the highest concentration of total free amino acids, while PL fish had significantly less (F = 5.563, df = 3, *p* < 0.01). Significant variation in the level of free amino acids in the blood of fish on different dietary treatments was found for eight indispensable (arginine, histidine, lysine, methionine, threonine, valine, isoleucine, leucine) and eight dispensable amino acids (alanine, asparagine, GABA, glutamic acid, proline, serine, taurine, tyrosine; Fig. 1, Table S2). For many dispensible amino acids, the SPH, followed by the PHP fish groups had the highest blood concentration levels. However, in many of the indispensable amino acids PHP fish had the highest concentrations. PHP fish had significantly higher blood levels of the branched chain amino acids; valine, leucine and isoleucine, compared to PL and FM fish and significantly higher levels of iso-leucine compared to SPH fish.

### Gut morphology

**Figure 2:**
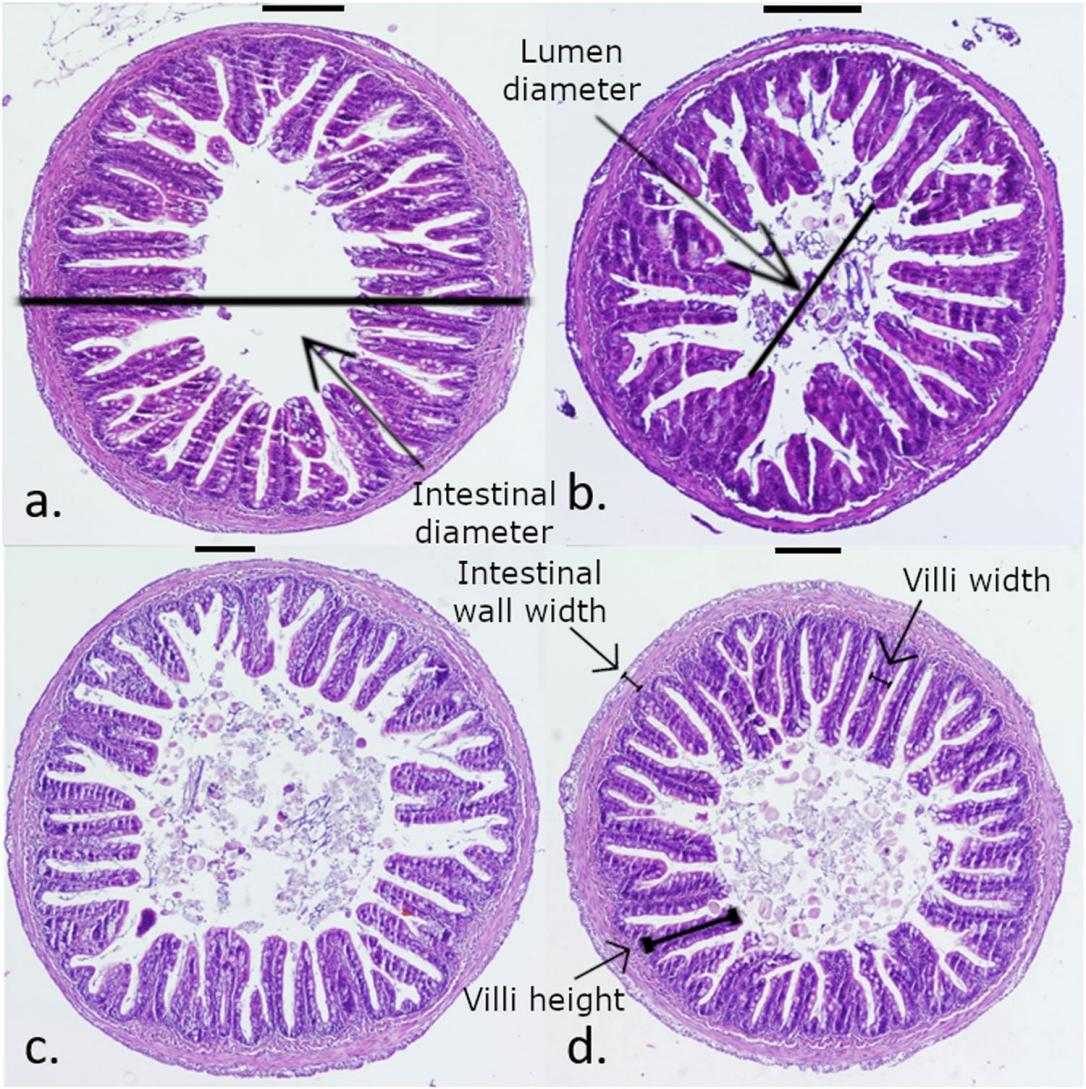
Example images of gut histology from fish fed four different treatment diets; (**a**.) FM, (**b**.) PL, (**c**.) PHP, and (**d**.) SPH. Blackline above each image represents scalebar = 200 µm. I.D. = intestine diameter, L.D. = lumen diameter, I.W.W. = intestinal wall width, V.H. = villi height, V.W. = villi width.

None of the fish evaluated in the present study showed signs of inflammation or enteritis in the gut (Fig. 2). SPH fish were found to have significantly wider villi (91.67 ± 20.33 μm) and wider intestinal walls (84.93 ± 23.62 μm) compared to FM fish (76.31 ± 14.92 μm and 58.97 ± 20.18 μm), PL fish (76.44 ± 22.63 μm and 63.18 ± 29.97 μm) and PHP fish (75.09 ± 14.89 μm and 65.57 ± 23.16; F = 2.224, df = 8, *p* < 0.05). No significant differences were found for the other measurements (villi height, Vh/Vw ratio, intestine diameter, interior lumen area, internal perimeter, external perimeter and perimeter ratio) between the different dietary groups (*p* > 0.05; Table 4).

**Table 4:** Gut histological morphometrics of salmon parr fed four different experimental diets for 12 weeks (FM, PL, PHP and SPH diets). Values with different superscripts in the same row are significantly different (*nested or ^†^ one-way ANOVA; p < 0.05, *n*=3, ± SD).

	FM	PL	PHP	SPH
Villi height (μm)*	**473.67** ± 130.92	**448.30** ± 227.62	**435.12** ± 159.02	**514.30** ± 161.04
Villi width (μm) ^†^	**76.31** ± 14.92^a^	**76.44** ± 22.63^a^	**75.09** ± 14.89^a^	**91.67** ± 20.33^b^
Vh/Vw ratio ^†^	**6.37** ± 1.97	**6.21** ± 3.35	**5.83** ± 1.80	**5.68** ± 1.60
Intestinal wall width (μm) ^†^	**58.97** ± 20.18^a^	**63.18** ± 29.97^a^	**65.57** ± 23.16^a^	**84.93** ± 23.62^b^
Intestine diameter (mm)*	**2.23** ± 0.56	**2.10** ± 0.84	**2.08** ± 0.50	**2.43** ± 0.54
Interior lumen area (mm^2^)*	**1.90** ± 1.18	**1.63** ± 1.30	**1.46** ± 0.68	**1.68** ± 0.71
Internal perimeter (mm)*	**24.73** ± 10.35	**21.13** ± 9.46	**25.16** ± 9.29	**27.15** ± 7.42
External perimeter (mm)*	**7.35** ± 1.88	**6.59** ± 2.47	**6.51** ± 1.50	**7.48** ± 1.50
Perimeter ratio (abs) *	**3.34** ± 0.81	**3.24** ± 0.88	**3.77** ± 0.62	**3.62** ± 0.55

### Gut microbiota composition

After quality filtering, there were a total of 7,910,986 reads, with reads per sample ranging from 31,266–205,661. Joining efficiency was 83.66 ± 0.14% and after sequence clustering, 738 operational taxonomic units (OTUs) were identified.

**Figure 3:**
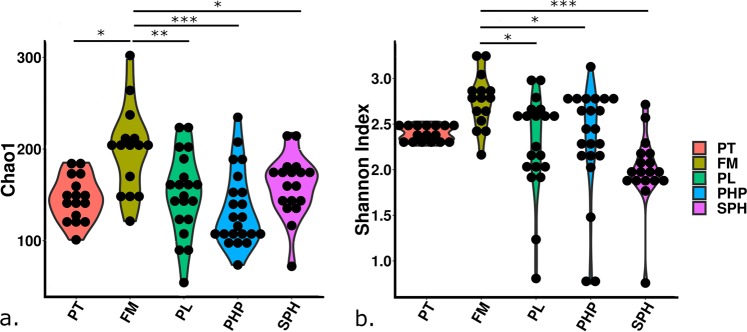
α-Diversity measures; (**a**) Chao1 index and (**b**) Shannon index, of 16S microbiota gut community compositions of pre-treatment salmon parr (PT) and fish that were fed four different dietary treatments (FM, PL, PHP and SPH) for 12 weeks. Significant differences denoted as: *(*p* < 0.05), **(*p* < 0.01), and ***(*p* < 0.001), n = 3.

Significant differences in α-diversity were found particularly between FM and the other treatment groups (Fig. 3a,b). Chao1 diversity and Shannon indices were used to calculate species richness in the gut communities. In both indices, fish on the FM diet had a significantly higher diversity (*p* < 0.001). Using the Chao1 diversity index, PT fish had a significantly lower diversity compared to fish on the FM diet (*p* < 0.05, Fig. 3a). However, this difference became insignificant using the Shannon index (Fig. 3b). Using the Shannon index, diversity of gut microbiota was significantly, positively related to weight gain (*r*_*s*_ = 0.588, *p* < 0.05). However, this relationship was not maintained when considering the Chao1 diversity index.

**Figure 4:**
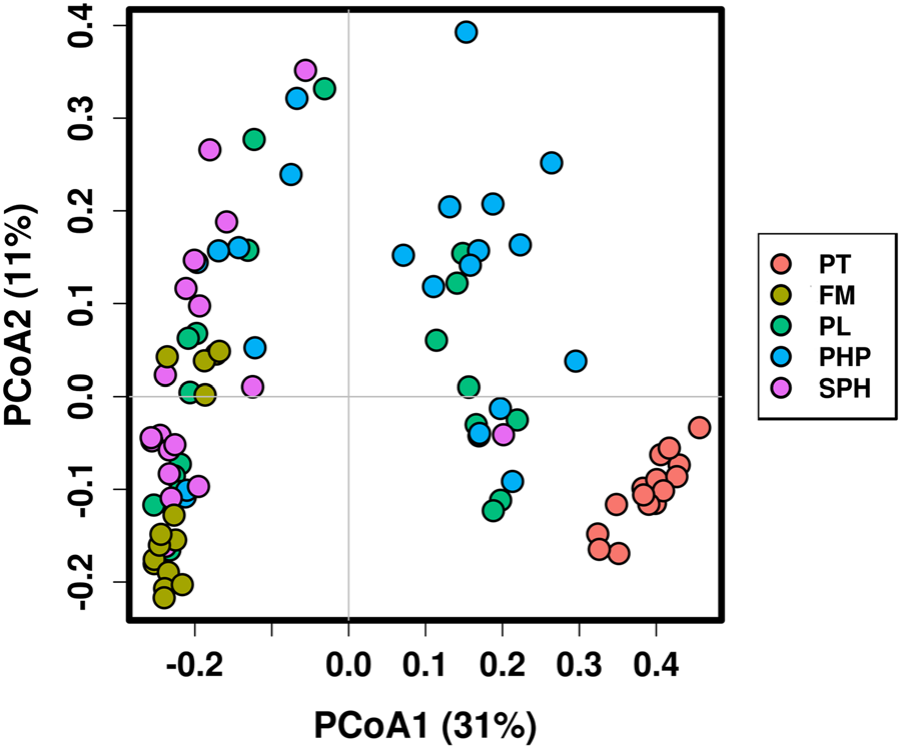
PCoA Bray Curtis plot of 16S OTU relative abundances associated with pre-treatment salmon parr (PT) and fish that were fed four different dietary treatments (FM, PL, PHP and SPH) for 12 weeks (*n* = 3). The first and second principal component explained 42% of the sample variations.

β-diversity was visualised using a PCoA Bray Curtis plot and significant separation of the gut microbial compositions by treatment was found (PERMANOVA, R^2^ = 0.306, *p* < 0.001; Fig. 4). The samples from PT fish separated furthest from the other groups and were tightly grouped. Samples from fish fed the FM diet also grouped close to each other, whereas the other three groups showed greater dispersion.

**Figure 5:**
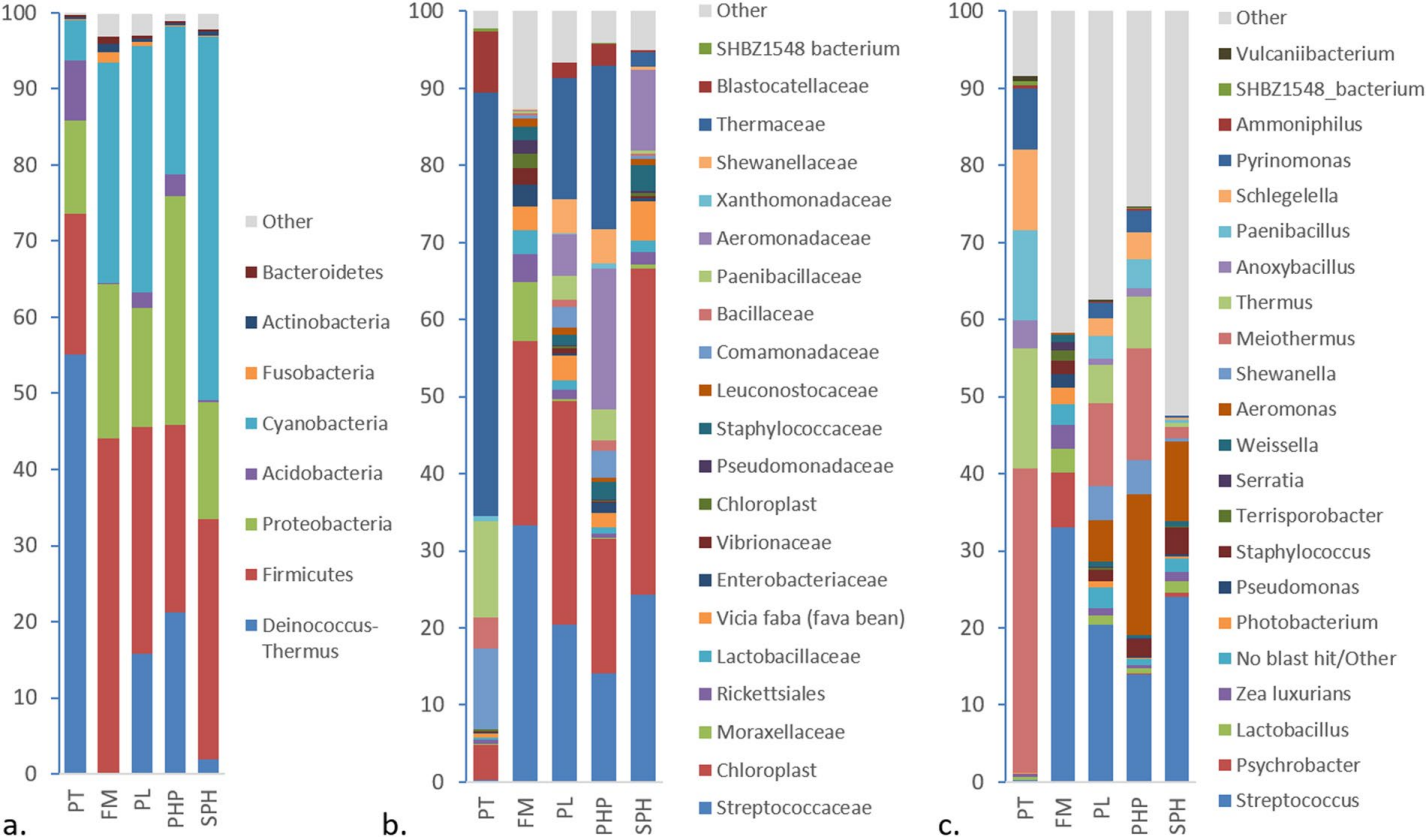
16S OTU relative abundance at the (**a**) phylum, (**b**) family and (**c**.) genus level for pre-treatment salmon parr (PT) and fish that were fed four different dietary treatments (FM, PL, PHP and SPH) for 12 weeks (*n* = 3).

The microbial community compositions of the intestinal contents of the salmon parr were assigned to 14 phyla. However, >90% of OTUs belonged to just five of these phyla. The relative abundance of the phyla identified from fish before the start of the dietary treatments was notably different from that of the fish at the end of the 12-week dietary intervention (Fig. 5a). PT fish were dominated by the phylum Deinococcus-Thermus (55%), followed by Firmicutes (19%) and Proteobacteria (12%). After the dietary treatments, all fish had significantly higher relative abundances of Firmicutes (*p* < 0.05) and Cyanobacteria (*p* < 0.001) and lower Deinococcus-Thermus (*p* < 0.001). The high abundance of Cyanobacteria observed possibly originated from the surrounding water or food and has been reported at similar levels in other studies investigating the gut microbiota of freshwater fish^47^. The relative abundance of Proteobacteria did not change significantly, although it was highest in PHP followed by FM fish (Fig. 5a). The community composition of FM and PT fish differed most significantly. FM fish also had a significantly higher ratio of Spirochaetes compared to fish fed the other three diets high in plant protein. All treatment groups were unique, separating significantly on the β-diversity PCoA plot. However, it can be seen in Fig. 4 that FM and SPH fish and, PL and PHP fish cluster more closely, indicating that the community composition was most similar between these treatment groups.

Analysis of the 16S data at family level showed that there was a core microbiota (OTUs present in ≥85% of samples^30^) shared across all treatment groups which involved *Streptococcaceae, Lactobacillaceae* and *Comamonadaceae* (Fig. 5b). *Thermaceae* were also in the core microbiota of PT, PL and PHP fish, and were found in 80% and 78% of FM and SPH fish, respectively. While *Bacillaceae* were in the core microbiota of PT, FM and PHP fish and were found in 80% of PL and SPH fish. PT fish had the most diverse core microbiota, which also included *Paenibacillaceae, Xanthomonadaceae, Blastocatellaceae* (Subgroup 4) and SHBZ1548 uncultured bacterium. FM fish also had the additional bacterial families; *Vibrionaceae* and *Peptostreptococcaceae* in their core microbiota. Fish fed the FM diet had a significantly higher ratio of the families *Moritellaceae, Psychromonadaceae, Helicobacteraceae* and *Bacteroidaceae* compared to fish fed the other three diets high in plant protein.

At genus level PT fish were dominated by only six genera; *Meiothermus*, *Thermus*, *Anoxybacillus*, *Paenibacillus*, *Schlegelella* and *Pyrinomonas* (Fig. 5c). These genera remained part of the core microbiota for fish on the PL and PHP diet. The gut microbiota of fish post-dietary treatments were all dominated by the genus *Streptococcus* and fish fed diets high in plant protein also contained high relative abundances of *Aeromonas* (Fig. 5c). Comparing relative abundance of lactic acid bacteria, fish in the PT group had significantly lower levels compared to the dietary treated fish, while FM fish had significantly more compared to fish on diets high in plant protein (*p* < 0.001).

**Figure 6:**
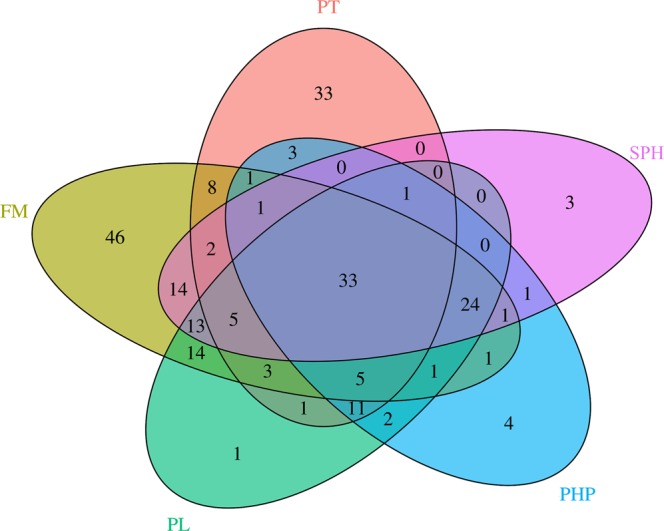
Total number of shared OTUs present in the intestinal contents of all fish in the pre-treatment (PT) and 4 dietary treatment (FM, PL, PHP and SPH) groups.

Fifty-seven OTUs were common to all groups post dietary treatment (Fig. 6). Of these, 33 were also found in PT fish. Nearly half of the shared OTUs were from the phylum Firmicutes, of which *Streptococcus* (PT 0.3%; FM 33.0%; PL 20.4%; PHP 14.0%; SPH 24.1%), followed by *Lactobacillus* (PT 0.4%; FM 3.1%; PL 1.2%; PHP 0.7%; SPH 1.5%) were the most commonly identified genera. *Meiothermus* (PT 39.5%; FM 0.01%; PL 10.7%; PHP 14.6%; SPH 1.4%) and *Aeromonas* (PT 0.003%; FM 0.2%; PL 5.4%; PHP 18.3%; SPH 10.4%) were also found at high relative abundances in most of the groups.

Comparing the genera present in the dietary groups, PL fish had only one genus (*Devosia*) that was at a significantly different (higher) relative abundance compared to all other fish. PHP and SPH fish contained five and three genera, respectively, at a significantly different relative abundance compared to all other fish. FM fish had a significantly higher relative abundance of 16 bacterial genera compared to the other three diets high in plant protein.

In the shared microbiota, found in all fish, there were several genera and species identified which, to the best of our knowledge, from reviewing published literature, have not been previously found in fish. These include *Enterococcus timonensis*, *Lactobacillus salivarius* and *Terrisporobacter sp*. These OTUs were not detected in the sequenced negative control.

To summarise, younger PT fish harbour a diverse community that is dominated by the phylum Deinococcus-Thermus and show low inter-sample variation. After the 12-week dietary treatment Firmicutes became the dominant phylum. FM fish had the highest α-diversity, while greater variation in inter-sample diversity and community composition was seen in the fish under the other dietary treatments that had significantly higher plant-protein content.

### Cost comparison

Often the cost of supplements required to sufficiently replace the nutrients derived from marine-origin resources are costly and considered un-economically viable. A cost comparison was carried out as part of this study to compare the variable protein ingredients in each of the four diets. The cheapest feed was the PL diet at €432.90 metric tonnes^−1^ (MT). The protein in the FM and PHP diets cost a similar amount; €540.58 MT^−1^ and €541.56 MT^−1^, respectively, while the SPH feed was the most expensive at €675.19 MT^−1^ (Table 1).

## Discussion

This study has shown that reducing the fishmeal component of feeds, from 35% to 15%, in the place of plant proteins (PL diet), resulted in reduced growth in Atlantic salmon parr. However, partial replacement of fishmeal with partly hydrolysed FPH in a high plant protein diet (PHP diet) allowed similar growth performance compared to fish fed the control diet (FM diet). A previous study looking at fishmeal replacement in Atlantic salmon parr reduced fishmeal to 60% of dietary protein contribution without negative effects on growth^48^. At the seawater stage, partial replacement of fishmeal with soybean meal or pea protein concentrate was also found to produce fish of a similar size^49^. However, these diets still contained 83–88% fishmeal dietary protein^49^. In this study, we have successfully reduced fishmeal to 6% of dietary protein contribution without negative effects on growth. More recent studies testing similar levels of fishmeal replacement with plant proteins have reported reduced growth^50,51^. The difference in our study is the supplementation of the plant-based diets with FPH. A number of studies have looked at FPH supplementation of standard fishmeal diets in Atlantic salmon with mostly positive outcomes in growth^35,42,52^. There has also been success with FPH supplementation of plant protein diets containing only 5% fishmeal in Atlantic salmon at seawater stage^53^. The results of this study suggest that this is also possible for Atlantic salmon parr on-grown in freshwater.

Specific growth rate, feed conversion ratio and protein efficiency ratio results followed the trend of highest growth rates in FM and PHP fish. Interestingly, the hepatosomatic index was highest in PHP fish, and significantly greater than that of PL fish. Hepatosomatic index is the ratio of liver weight to body weight. It provides an indication of the status of energy reserve and protein accretion in the fish^23,54,55^. During salmon production, it is ideal to have salmon parr at maximum size and optimum health in advance of smoltification. During smoltification, a period of high energy requirements, whole-body and liver lipid and energy reservoirs become depleted^55,56^. Atlantic salmon parr with higher levels of stored lipids may have increased energy for smoltification^57^, which may, in turn, prevent a ‘protein sparing effect’^58^ and, ergo, result in healthier, larger smolts. Further testing is needed to determine whether the higher hepatosomatic index in the PHP dietary group could enhance robustness during smoltification, e.g. survival rate, disease susceptibility and meeting energy/lipid depletion demand.

The fish fed the PL diet did not show a difference in feed intake but grew significantly less than the other dietary groups (4.6–11.7% smaller fish at the end of dietary intervention). The blood concentration of total free amino acids in these fish was the lowest of all the groups (5.8–17.8% less). These results suggest that reduced digestibility and anti-nutritional factors related to the high level of soybean meal protein concentrate in the PL diet resulted in reduced blood amino acid concentrations and growth performance. Similar results have been reported elsewhere^59,60^. It was noted that although many of the blood free amino acids in the PL fish were significantly lower than those recorded in PHP and SPH fish, this was not the case for FM fish. Hence, plant-related, anti-nutritional factors may have also inhibited the absorption of other nutrients, such as macro and trace metals, and lipids, abetting growth inhibition, as seen previously^61–64^.

Protein requirements are related to amino acid bioavailability and, it has been shown here and in numerous previous studies that plant-derived protein is of inferior quality in terms of digestibility and bioavailability compared to fishmeal^5,21,65–67^. However, our results have shown that supplementation of a predominantly plant protein diet, containing only 5% fishmeal, with partly-hydrolysed FPH (PHP supplement) is as effective as a 35% fishmeal diet. Indeed, despite consuming similar levels of feed, fish on the PHP diet were significantly heavier and had a better condition factor compared to PL fish and as well as FM fish (in terms of final weight only for the latter). FPH is considered an excellent ingredient for aquafeeds due to its nutritional value and functional and bioactive properties^68^. Their nutritional and health-promoting characteristics are due to their significant fractions of single amino acids and low-molecular-weight peptides that are generally easier to digest and absorb. Interestingly, it is now known that many amino acids are more rapidly and efficiently absorbed as di- and tripeptides, rather than single amino acids^69^. In this study, although SPH fish had, overall, the highest concentration of total free amino acids in their blood, PHP fish had the highest levels of essential amino acids. The blood levels of branched chain amino acids of PHP fish were at a significantly higher concentration than FM and PL fish and this remained true for SPH fish in terms of isoleucine. Branched chain amino acids play important structural roles and act as an anabolic signal for protein synthesis^70^. Increased bioavailability of essential amino acids, especially branched chain amino acids, as indicated by blood amino acids concentrations, appear to have stimulated increased protein synthesis and growth in the fish. This finding has been reported in Atlantic salmon at different life stages and in other aquaculture species also^21,23,35,42,52,71,72^. However, studies have found that results are dose-dependent and negative effects can occur from higher levels of FPH inclusion^35,52,53^. Although the PHP and SPH ingredients were supplemented at the same concentration of total feed, the lower molecular weight proteins that make up the SPH may have meant it acted similar to a higher dose of hydrolysate, and therefore, like previous studies, resulted in reduced effectiveness for growth stimulation. Whole-body protein levels were lowest in SPH fish, which was significantly less compared to that of FM fish. This result confirms that the dietary protein in the SPH diets was not fully assimilated. The mechanistic reasons behind these outcomes warrant further investigation. In comparison to previous studies, the use of protein hydrolysate can influence body composition in the fish. For example, the feeding of shrimp protein hydrolysate to Nile tilapia (*Oreochromis niloticus*) had decreased body protein level as the inclusion rate were increased^73^. Similar results were also reported in turbot when fed a high plant protein diet and increasing levels of fish protein hydrolysate^74^.

FM fish did not show as high levels of blood amino acids as PHP fish (3174 ± 287 vs. 3357 ± 298 µg mL^-1^), however, their growth performance (FI, FCR, SGR, PER, HSI and final weight) was on par. It has been reported previously that fishmeal contains a unique array of growth factors that support optimum growth in carnivorous species^2,3^. This suggests that alternative pathways, beyond protein absorption, were stimulating high growth rates in these fish.

Gut morphology and gut microbial community composition were investigated. Inflammatory or degenerative changes in the gut, indicative of soy-induced enteritis, were not present in any histological section from the fish examined. The only significant differences recorded were wider villi and thicker intestinal walls in SPH fish. Larger villi (higher and wider) equate to more and/or bigger cells, providing greater surface area for absorption of nutrients and metabolites. This is supported by SPH fish having the highest level of total blood amino acids. It could be hypothesised that this morphological characteristic would promote growth. However, this was not seen here and similarly reported not to correlate in other species, elsewhere^75^.

16S rRNA sequencing revealed that fish from each dietary group had distinct gut microbial communities. β-diversity analysis of the microbiota in the intestinal contents of the fish revealed a clear and significant separation of FM fish from fish on the other three diets high in plant protein. Dietary protein source has previously been reported to alter gut microbiota composition^4,10^. Zarkasi *et al*.^76^ compared the microbiota of Atlantic salmon fed a commercial (35% fishmeal) diet and diets that were low in protein or low in fishmeal. Similar to this study, their β-diversity plots show the pre-treatment fish and the fish fed the commercial fishmeal diets clearly separating from the other experimental groups. This same trend was seen in another Atlantic salmon study by Gajardo *et al*.^4^.

Fish on the FM diet had significantly higher gut microbial α-diversity. Fifty-four OTUs were found only in this group and they also had a significantly higher relative abundance of 16 shared OTUs, primarily from the Proteobacteria and Firmicutes phyla. In adult humans and mammals, a diverse microbiota has frequently been linked with a balanced, well-functioning metabolism^77–79^. It has been shown in a number of studies that the gut microbiome affects metabolism in fish; affecting nutrient uptake^80^, metabolism pathways^81^ and ultimately growth^82^. In particular, having a diverse gut microbiome can maintain fish health through the stimulation of the fish innate immunity, production of antimicrobial compounds (e.g. bacteriocins, peptides and proteins) from invading pathogens, and depriving the gut surface area for pathogenic bacteria in establishing itself^83^. Furthermore, Webster *et al*.^84^, comparing the gut microbiota of Atlantic salmon parr sourced from hatcheries and from the wild, found, overall, wild populations had considerably higher microbial diversity than hatchery populations. The interaction of the gut microbiota with dietary components is complex and multifaceted. However, it is likely that the increased microbial diversity recorded in the intestinal contents of FM fish enhanced nutrient uptake and metabolism beyond that of protein absorption. Furthermore, it was found that PL fish had high relative abundances of *Aeromonas* bacteria. This genus includes opportunistic pathogenic bacteria which may have played a role in reducing the growth performance in this group^85^.

Comparing the gut microbial composition of fish fed the diets high in plant protein to the fishmeal control diet, bacterial families known for their proteinaceous metabolic activity (e.g. *Vibrionaceae, Peptostreptococcaceae, Pseudomonadaceae, Moraxellaceae*) were reduced in the place of increased carbohydrate or broad range metabolisers (e.g. *Thermaceae, Shewanellceae, Aeromonadaceae*). Interestingly, these alterations were not as significant in fish supplemented with the SPH. Thus, data suggests that the gut bacterial community adapted to the different dietary proteins received. *Moraxellaceae* as well as *Pseudomonadaceae*, among others, were the most commonly found microbial families in captive parr^86^. *Moraxellaceae* has, thus far, not been frequently reported as a significant member of Atlantic salmon gut microbiota, however, a recent study found that it increased in relative abundance in Atlantic salmon administered antibiotics^87^. *Peptostreptococcaceae, Pseudomonadaceae* and *Shewanellceae* have been reported in previous studies investigating the gut microbiota in Atlantic salmon parr^27,28,88^ while *Thermaceae* has previously been associated with earlier life stages^89^. Interestingly, certain strains of *Shewanella sp*. have been used successfully as probiotics to improve growth in Senegalese sole (*Solea senegalensis*)^90^ while *Aeromonas sobria* administered to rainbow trout (*Oncorhynchus mykiss*) improved immunity and survival when fish underwent bacterial pathogen challenges^91^. A recent study investigating the effect of alternative dietary protein sources on the gut microbiota of post-smolt Atlantic salmon found that replacement of fishmeal with sunflower extract caused a decrease in *Peptostreptococcaceae* while replacement with pea protein resulted in an increase in *Vibrionaceae*^10^. These results highlight the varied effects that even different plant protein sources can have on gut microbial communities.

There have only been a few studies investigating the gut microbial composition of Atlantic salmon at the freshwater stage^27,28,30,88^. From these studies, core or principal phylotypes reported included Firmicutes, Proteobacteria, Actinobacteria and Tenericutes, as well as Clostridiales, *Mycoplasmataceae, Enterobacteriaceae, Comamonadaceae, Ruminococcaceae, Microbacteriaceae, Hyphomicrobacteriaceae, Peptostreptococcaceae, Yersinia, Vagococcus, Acinetobacter, Shewanella, Microbacterium, Cellulomonas, Serratia, Pseudomonas, Chryseobacterium, Staphylococcus, Escherichia/Shigella, Brucella* and *Corynebacterium* among others. In this study, we also report significant levels of Firmicutes, Proteobacteria, *Enterobacteriaceae, Comamonadaceae, Peptostreptococcaceae* and *Shewanella*. Notably absent are significant levels of Tenericutes, *Mycoplasmataceae* and *Pseudomonas*, microbes that have been previously reported in other studies investigating the gut microbiota of Atlantic salmon (parr)^27,29,30,92^. The small number of studies in this area to date and the wide variation in results reported means that what constitutes a healthy microbiota for Atlantic salmon parr has yet to be fully defined. Without this knowledge, it is difficult to accurately determine the effects of dietary treatment.

In conclusion, FPH can be produced from fish processing by-products, fishery by-catch, and low-value pelagic species not currently directly consumed by humans. When using sustainable processing practices, it can be one of multiple product streams derived from using the entire fish. Its high nutritional value allows it to be used in small quantities as a supplement to fortify diets. These characteristics suggest that its use could go some way towards sustainable food production and help reduce the volume of wild fish species used in aquafeeds, an important consideration in light of recent biodiversity reports^93^. This study has shown that farmed Atlantic salmon parr can grow successfully on an 80% plant protein diet when supplemented with a partly-hydrolysed FPH (PHP). PHP fish also had relatively high heptosomatic indices, possibly indicating higher liver lipid stores that would benefit fish during smoltification. Furthermore, a cost comparison of the different feeds highlighted that this formulation is an economically viable alternative. The results indicate that improved essential amino acid bioavailability, in particular branched chain amino acids, facilitated the high growth rates recorded in PHP fish. Although the study reports some significant results, not all growth performance indicators revealed statistically significant differences. Further research into the effects of the different diets over extended periods, and associated variations in gut microbiota using metabolomics and shotgun sequencing to ascribe digestive roles would be beneficial to gain a greater understanding of the interaction of dietary nutrients and gut microbiota and their effects on host health, development and growth.

## Materials and methods

### Experimental diets

The tested fish protein hydrolysates (Biomarine Ingredients Ireland Ltd., Monaghan, Ireland) were produced from whole blue whiting, *Micromesistius poutassou*, frozen directly after catching in the north-east Atlantic. Following thawing, lipids and bone were removed from the blue whiting and the remaining raw material was enzymatically hydrolysed. The water-soluble protein hydrolysate fraction (SPH) and the insoluble partly hydrolysed protein fraction (PHP) were separated and spray-dried to prevent thermal damage to the protein. The SPH hydrolysate was composed of lower molecular weight peptides and single amino acids and contained 91% protein, of which 96% was soluble. The PHP hydrolysate was composed of low and medium molecular weight peptides and contained 68% protein, of which 18% was soluble (Table S1).

Four diets were formulated and manufactured by the Aquaculture Nutrition and Aquafeed Research Unit (ANARU) at Carna Research Station, Ryan Institute, National University Ireland Galway, Ireland. Commercially available feed ingredients were used and diets were formulated to meet the dietary requirements of appropriately sized salmon, including vitamin and mineral requirements^70^. Formulation and proximate composition of experimental diets are shown in Table 1. Diet A (FM), the control diet, was formulated to replicate fishmeal-based commercial salmon aquafeeds, containing 35% fishmeal. The other three diets were high in plant protein (80%) and low in fish-derived protein. Diet B contained 15% fishmeal (PL), Diet C contained 5% fishmeal and 10% partly hydrolysed fish protein (PHP) and Diet D contained 5% fishmeal and 10% soluble protein hydrolysate (SPH). All diets were iso-nitrogenous and iso-lipidic in content (Table 1). The total amino acid content of the four diets and the PHP and SPH supplement are outlined in Table 5. Yttrium oxide was included in all experimental diets to allow for apparent digestibility analyses. The experimental diets were extruded (1 and 2 mm pellets) in a single screw extruder (PM-80, Bottene, Vicenza, Italy) and dried at 40 °C in a dehumidifying oven. There were no differences in physical quality or sinking properties of the four diets.

**Table 5:** Total amino acid composition of the two supplementary protein ingredients (% of powder), the four treatment feeds and the essential amino acid requirements of Atlantic salmon^70^ (% of diet dry matter; ND = not determined).

	Supplements		Diet				Atlantic salmon
	PHP	SPH	FM	PL	PHP	SPH	(av. 0.2–500 g)
Arginine	4.63	5.71	3.03	3.18	3.11	3.15	1.81
Histidine	1.62	1.17	1.41	1.39	1.26	1.38	0.80
Isoleucine	3.66	3.01	2.12	2.14	2.16	2.02	1.32
Leucine	5.91	5.63	3.75	3.79	3.73	3.65	2.31
Lysine	6.48	8.51	3.57	3.16	3.10	3.25	2.55
Methionine + Cystine	3.14	2.67	2.08	1.75	1.81	2.04	1.29
Phenylalanine + Tyrosine	6.76	3.92	3.63	3.90	3.86	3.56	2.70
Threonine	3.63	3.92	1.88	1.85	1.81	1.75	1.58
Tryptophan	ND	ND	ND	ND	ND	ND	0.36
Valine	4.12	4.05	2.34	2.39	2.37	2.26	1.77
Cystic acid	ND	ND	0.87	0.94	1.10	0.99	
Taurine	ND	ND	0.39	0.19	0.27	0.32	
Asparagine	7.55	8.28	4.58	4.71	4.88	4.77	
Serine	3.66	4.11	2.20	2.23	2.35	2.26	
Glutamic acid	9.59	15.00	9.39	9.42	10.09	9.63	
Glycine	3.75	6.51	2.61	2.06	2.24	2.29	
Alanine	4.54	5.93	2.42	2.04	2.12	2.18	
Proline	2.84	3.70	2.61	2.54	2.77	2.60	

### Fish and rearing conditions

The 12-week feeding trial was carried out at Salmon Springs Ltd. freshwater juvenile salmon rearing facility in Co. Galway, Ireland. Atlantic salmon (*Salmo salar*) were raised from eggs (donated kindly by Stofnfiskur, Iceland) on site. Prior to the experiment, the fish were fed commercial diets (Skretting UK, Cheshire, UK). At the start of the experiment, salmon (8.44 ± 0.78 g, F = 1.567, df = 11, p = 0.103) were randomly distributed into 1 m^3^ fibreglass tanks (at a density of 6.5 kg m^−3^ in 0.4 m^3^ of water, *n* = 3). The triplicate tanks were at an initial density of 16.2 kg L^−1^. Average tank density for all treatment groups had reached 20 kg L^−1^ by day 40 of the feeding trial and tanks were maintained at this density, by periodic removal of fish, for the remainder of the trial. The tanks were set up on a flow-through system and supplied with natural spring freshwater at a flow rate of 4 L min^−1^ and further aerated with air pumps. A natural photoperiod was used which ranged from 14.75–16.75 h daylight. The average water temperature during the trial was 11.2 ± 0.6 °C and dissolved oxygen level was recorded throughout (9.1 ± 0.71 mg L^−1^). Triplicate groups of fish were fed one of the four treatment diets via automatic feeders during daylight hours (~1.5% BW) for 12 weeks. Tank weights were measured fortnightly to allow for feed adjustments. Feeding was withheld 24 hr prior to final morphometric measurements at the end of the trial, to ensure that fish were clear from residue feed. All experiments were approved by the Ethics Committee of University College Cork, licenced by HPRA, Ireland (project authorisation AE19131/P068) and in full accordance with the European Community Council Directive (86/609/EEC).

### Sample collection and analyses

#### Sample collection

Fish were sampled the day before the dietary treatment began (pre-treatment group, PT) and at the end of the trial. Fish taken for biological samples were not starved in advance of sampling to ensure collection of intestinal contents and reduce possible alterations of blood amino acid concentrations. From each tank, eight random fish were sampled, euthanised and blood was taken immediately from the caudal vein with a heparinised 25 G needle and 1 mL syringe. Blood samples and carcasses were stored at −20 °C until analysis.

A further eight fish/ tank were culled and dissected to collect organs. The gastrointestinal tract from the stomach to the anus was removed from the peritoneal cavity, placed on a sterile petri dish and aseptically dissected. The intestinal contents from the distal intestines were frozen immediately on dry ice before transferring to −80 °C storage for later analysis. Livers were removed and weighed.

Fish remaining in the tanks after collection of biological samples were starved for 24 h to standardise gut contents. Subsequently, the wet weight and forktail length of each individual fish from each tank were measured.

#### Proximate composition and amino acid determination

Feed ingredients, test diets and fish body proximate composition were determined using AOAC (2002)^94^ methods. Whole carcasses from each tank were pooled in duplicate and homogenised (Robot Coupe blixer 2 commercial food processor, Stephens Catering Equipment Co. Ltd., Ireland) to create a uniform mince. Moisture content was determined gravimetrically after drying at 105 °C for 24 h. Ash content was measured by incinerating the samples at 550 °C for 16 h. Total nitrogen content was determined by the Kjeldahl method and crude protein content was estimated by multiplying total nitrogen content by 6.25 conversion factor. Lipid content was determined gravimetrically after extraction following a modified Bligh and Dyer method^95^.

Whole blood samples were used for the analysis of free amino acid content according to the methods of McDermott and colleagues^96^. Amino acids were quantified using a Jeol JLC-500/V amino acid analyser (Jeol (UK) Ltd., Garden city, Herts, UK) fitted with a Jeol Na+ high-performance cation exchange column. For total amino acid content of feeds, proteins were hydrolysed in 6 N HCl at 110 °C for 23 h and the resulting hydrolysates analysed as per free amino acid method.

#### Intestinal morphology

At the end of the feeding trial, three fish were sampled from each tank and the posterior intestine was fixed in 10% neutral buffer formalin for histological processing. Samples were subsequently dehydrated and embedded into wax for sectioning. Sample sections were stained using Mayer’s Haematoxylin and Eosin (Thermo Fisher Scientific, Waltham, Massachusetts, US). Measurements of the gut were carried out using a light microscope and processed using ImageJ^97^.

#### Gut microbiota 16S rRNA sequencing

DNA extraction from samples was performed using a QIAGEN QIAamp Fast DNA Stool Mini Kit (Qiagen Ltd, Manchester, England) according to the manufacturer’s protocol with the following modifications outlined by Dehler *et al*.^27^. Extracted DNA was quantified by NanoDrop^TM^ spectrometry (Thermo Fisher Scientific, Waltham, Massachusetts, US). The V3-V4 variable region of the 16S rRNA gene was amplified from the DNA extracts using the Illumina 16S metagenomic sequencing library protocol. The PCR reactions were performed in a 25 μL reaction volume containing DNA template, 12.5 μL Biomix Red (Bioline, Memphis, USA), 5 μL each of forward and reverse primers (1 μM), and PCR grade water to final volume. PCR amplification conditions included initial denaturation at 95 °C for 5 min, followed by 35 cycles of denaturation at 95 °C for 30s, annealing at 55 °C for 30s, and extension at 72 °C for 5 min. PCR products were cleaned using AMPure XP magnetic bead-based purification (Beckman Coulter Life Sciences Brea, California, United States). This was followed by indexing PCR, which attached Nextera XT barcodes and Illumina® sequencing adapters to the 5′ overhangs and another round of AMPure XP clean-up. After quantifying the samples, using Invitrogen Qubit 4 Fluorometer and high sensitivity DNA quantification assay kit (Thermo Fisher Scientific, Waltham, Massachusetts, US), they were pooled in an equimolar fashion. The pooled sample was run on the Agilent Bioanalyser for quality analysis prior to sequencing. Samples were sequenced on the MiSeq sequencing platform at the Teagasc Sequencing Facility, Cork, Ireland, using a 2 ×300 bp cycle kit, following standard Illumina® sequencing protocols.

#### Bioinformatics analysis

Three hundred base pair paired-end reads were assembled using FLASH. The QIIME suite of tools, v1.8.0, was used for further processing of paired-end reads, including quality filtering based on a quality score of >25 and removal of mismatched barcodes and sequences below length thresholds^98^. De-noising, chimera detection and operational taxonomic unit (OTU) grouping at 98% similarity were performed using USEARCH v7 (64-bit)^99^. Taxonomic ranks were assigned by the alignment of OTUs using PyNAST to the SILVA SSURef database release 128^100^. Alpha and beta diversities were calculated using QIIME on weighted Unifrac distance matrices.

16S microbiota data was entered into Calypso^101^ for further analysis and statistical testing. Principal co-ordinate analysis (PCoA) plots were visualised using Bray-Curtis calculated distances and differences between dietary treatments were determined using permutational multivariate analysis of variance (PERMANOVA-Adonis). The Benjamini-Hochberg adjustment procedure was applied with the false discovery rate (FDR) set at 20% to correct for multiple testing.

### Calculations and statistics

Apparent digestibility coefficient of dry matter (ADC DM), specific growth rate (SGR), feed conversion ratio (FCR), protein production value (PPV), protein efficiency ratio (PER) and hepatosomatic Index (HSI) were expressed as the following:

$$ADC\,DM\,( \% )=(1-(\frac{marker\,in\,feed}{marker\,in\,faeces}))\times 100$$

$$SGR\,( \% )=\frac{ln{W}_{2}-ln{W}_{1}}{Feeding\,days}\times 100$$

4.4.3 $$FCR=\frac{Feed\,consumed/fish}{{W}_{2}-{W}_{1}}$$

4.4.4 $$PPV=\frac{({W}_{2}\times C{P}_{2}\,-{W}_{1}\times C{P}_{1})}{Protein\,consumed}$$

4.4.5 $$PER=\frac{{W}_{2}-{W}_{1}}{Protein\,consumed/fish}$$

4.4.6 $$HSI( \% )=\frac{{W}_{l}}{{W}_{f}}\times 100$$

Where, W_1_ and W_2_ are average initial and final fish weights, respectively; CP_1_ and CP_2_ are the crude protein of the fish at the beginning and end of the feeding trial, respectively; W_l_ is the weight of liver and W_f_ is the weight of fish at the time of sampling and ln is the natural logarithm.

Statistical analyses were performed using Microsoft Excel® and the SPSS® computer programs (SPSS Statistical Software, Inc., Chicago, IL.). All data were subjected to Analysis of Variance (ANOVA), or non-parametric alternative where appropriate, and pairwise comparisons were conducted by Tukey’s test. Spearman’s correlation was carried out on weight gain in relation to the alpha diversity indices; Shannon and Chao1. Gut morphometrics were compared between treatment groups using anested ANOVA. The significance level was determined at the 95% probability level (p < 0.05). GraphPad Prism Software (GraphPad Software, San Diego, CA, USA) was used for the generation of figures.

A cost comparison of the diets was made by comparing the cost of the variable protein ingredients; fishmeal, SPC and the experimental protein supplement hydrolysates; PHP and SPH. The value for the fishmeal was taken from the website indexmundi.com, a website providing detailed country statistics, charts, and maps compiled from multiple sources. The source reported was the World Bank. The cost for SPC was taken from the website Alibaba.com, a global wholesale commodities website.

## Supplementary information

Supplementary Tables.
