# Supplementary material for: Replacing fishmeal with plant protein in Atlantic salmon (Salmo salar) diets by supplementation with fish protein hydrolysate
Source: Sci Rep. 2020 Mar 6;10:4194. doi: 10.1038/s41598-020-60325-7 (PMC7060232; doi:10.1038/s41598-020-60325-7)
Supplement: Supplementary file 1 — Supplementary Tables. [file 41598_2020_60325_MOESM1_ESM.docx]

Replacing fishmeal with plant protein in Atlantic salmon (*Salmo salar*) diets by supplementation with fish protein hydrolysate

Egerton, S.^1,2,3^, Wan, A.^4^, Murphy, K. ^3,5^, Collins, F.^1,3,5^, Ahern, G. ^1,3,5^, Sugrue, I. ^1,3,5^, Busca, K. ^3,5^, Egan, F.^2^, Muller, N.^4^, Whooley, J.^6^, M^c^Ginnity, P.^2^, Culloty, S.^2,7^, Ross, P.^1,3,5^, Stanton, C.^3,5*^

# Supplementary Tables

Table S1. Molecular weight profile of the soluble protein fractions of the partly hydrolysed protein hydrolysate powder (PHP) which contained 18% soluble protein and the soluble protein hydrolysate powder (SPH) which contained 96% soluble protein, used as experimental ingredients to supplement high plant diets for Atlantic salmon parr; kDa = kilo-Daltons.

|  | % of Soluble Protein | | % of Total Protein | |
| --- | --- | --- | --- | --- |
|  | PHP | SPH | PHP | SPH |
| >20kDa | 0.23 | 0.04 | 0.04 | 0.04 |
| 20-10kDa | 0.49 | 0.26 | 0.09 | 0.25 |
| 10-5kDa | 1.97 | 1.36 | 0.35 | 1.31 |
| 5-2kDa | 6.94 | 6.09 | 1.25 | 5.85 |
| 2-1kDa | 13.5 | 12.89 | 2.43 | 12.37 |
| 1-0.5kDa | 27.7 | 27.35 | 4.99 | 26.26 |
| <0.5kDa | 49.18 | 52.01 | 8.85 | 49.93 |

Table S2. Blood amino acid concentrations (µg/mL) in fish fed one of four diets; FM, PL, PHP or SPH. Values are means ± SD (*n*= 3). Statistical analysis was completed using one-way ANOVA and Tukey’s Multiple Comparison test. Values with different superscripts in the same row are significantly different.

| **Amino acid** | **FM** | **PL** | **PHP** | **SPH** | **ANOVA (one-way)** |
| --- | --- | --- | --- | --- | --- |
|  |  |  |  |  | **p <** |
| Alanine | 236.9 ± 34.6 ^a^ | 265.6 ± 34.7 ^a^ | 307.1 ± 30.0 ^b^ | 314.6 ± 30.4 ^b^ | 0.001 |
| Asparagine | 54.0 ± 10.7 ^a^ | 71.3 ± 13.4 ^b^ | 65.3 ± 17.5 ^ab^ | 76.1 ± 11.3 ^b^ | 0.01 |
| Cysteine | 35.4 ± 7.7 | 34.6 ± 5.1 | 36.5 ± 4.1 | 39.5 ± 5.6 | ns |
| GABA | 22.5 ± 7.7 ^a^ | 15.8 ± 3.0 ^b^ | 13.2 ± 2.1 ^b^ | 13.5 ± 1.6 ^b^ | 0.001 |
| Glutamic acid | 215.0 ± 34.5 ^a^ | 238.7 ± 43.9 ^ab^ | 269.0 ± 32.8 ^b^ | 248.9 ± 26.0 ^ab^ | 0.05 |
| Glycine | 176.0 ± 19.1 | 174.7 ± 31.1 | 175.8 ± 19.2 | 198.7 ± 19.5 | ns |
| Proline | 140.8 ± 23.0 ^a^ | 152.1 ± 25.5 ^a^ | 180.8 ± 18.8 ^b^ | 194.1 ± 16.5 ^b^ | 0.001 |
| Serine | 104.2 ± 10.4 ^a^ | 103.7 ± 17.7 ^a^ | 113.8 ± 15.9 ^ab^ | 124.0 ± 8.9 ^b^ | 0.01 |
| Taurine | 1212.1 ± 153.2 ^a^ | 885.8 ± 140.3 ^bc^ | 913.5 ± 159.0 ^c^ | 1065.3 ± 92.5 ^ac^ | 0.001 |
| Tyrosine | 26.1 ± 4.4 ^a^ | 26.8 ± 8.3 ^a^ | 41.2 ± 10.0 ^b^ | 32.1 ± 4.4 ^a^ | 0.001 |
| Arginine | 116.1 ± 37.2 ^a^ | 131.6 ± 16.1 ^a^ | 152.9 ± 30.1 ^b^ | 155.5 ± 13.0 ^b^ | 0.01 |
| Histidine | 27.2 ± 4.1 ^a^ | 35.7 ± 7.0 ^b^ | 39.2 ± 9.2 ^b^ | 38.6 ± 4.1 ^b^ | 0.001 |
| Isoleucine | 38.3 ± 7.3 ^a^ | 42.0 ± 9.5 ^a^ | 61.4 ± 12.3 ^b^ | 46.3 ± 5.0 ^a^ | 0.001 |
| Leucine | 79.7 ± 13.4 ^a^ | 84.7 ± 18.2 ^a^ | 116.8 ± 22.5 ^b^ | 99.7 ± 10.8 ^ab^ | 0.001 |
| Lysine | 352.3 ± 112.1 ^a^ | 415.5 ± 57.4 ^b^ | 472.5 ± 47.5 ^bc^ | 505.5 ± 36.9 ^c^ | 0.001 |
| Methionine | 28.2 ± 6.9 ^ab^ | 20.5 ± 7.4 ^a^ | 28.6 ± 7.2 ^ab^ | 31.5 ± 8.3 ^b^ | 0.05 |
| Phenylalanine | 47.1 ± 12.0 | 45.6 ± 11.9 | 58.9 ± 23.2 | 57.8 ± 12.2 | ns |
| Threonine | 94.4 ± 11.2 ^a^ | 99.2 ± 16.2 ^a^ | 119.5 ± 17.6 ^b^ | 118.0 ± 9.4 ^b^ | 0.001 |
| Tryptophan | 41.6 ± 104.7 | 8.9 ± 4.0 | 9.5 ± 1.7 | 10.9 ± 2.2 | ns |
| Valine | 121.4 ± 12.3 ^a^ | 143.8 ± 25.2 ^ac^ | 177.2 ± 26.0 ^b^ | 158.0 ± 17.4 ^bc^ | 0.001 |
| Cystic acid | 4.8 ± 0.7 ^ab^ | 4.0 ± .9 ^a^ | 4.6 ± .7 ^ab^ | 5.1 ± 0.9 ^b^ | 0.05 |
| Total | 3174.2 ± 286.8 ^ab^ | 3000.8 ± 402.8 ^b^ | 3357.4 ± 298.1 ^ab^ | 3533.6 ± 205.3 ^a^ | 0.01 |
